# Proportions of Proinflammatory Monocytes Are Important Predictors of Mortality Risk in Hemodialysis Patients

**DOI:** 10.1155/2017/1070959

**Published:** 2017-10-22

**Authors:** Yachung Jeng, Paik Seong Lim, Ming Ying Wu, Tien-Yu Tseng, Chang Hsu Chen, Hung Ping Chen, Tsai-Kun Wu

**Affiliations:** ^1^Division of Biostatistics and Epidemiology, Department of Medical Research, Tungs' Taichung MetroHarbor Hospital, Taichung, Taiwan; ^2^Department of Internal Medicine, National Taiwan University Hospital, Taipei, Taiwan; ^3^Division of Renal Medicine, Tungs' Taichung MetroHarbor Hospital, Taichung, Taiwan; ^4^Department of Internal Medicine, Taipei Medical University, Taipei, Taiwan; ^5^Department of Rehabilitation, Jenteh Junior College of Medicine, Nursing and Management, Miaoli, Taiwan

## Abstract

Despite the continuous progression in dialysis medicine, mortality and the burden of cardiovascular disease (CVD) among hemodialysis patients are still substantial. Substantial evidence suggests that proinflammatory (CD16+) monocytes contribute to the development of atherosclerosis. A cohort of 136 stable hemodialysis patients (follow-up: 6.25 year) was assessed to investigate the association between the proportion of CD16+ monocytes for all-cause and CVD mortalities. The CD16+ monocytes were associated with both mortalities after adjusting for a preexisting CVD history. Compared to the reference group (CD16+ monocytes within [15.6–18.6], the first and second quartile), patients with CD16+ monocytes above the highest quartile level (>21.5) had an adjusted hazard ratio (HR) of 30.85 (95% confidence interval [CI]: 7.12–133.8) for CVD mortality and 5.28 (2.07–13.49) for all-cause mortality, and those with CD16+ monocytes below the lowest quartile ≤15.6), had significantly elevated death risks after 3.5-year follow-up (HR [95% CI]: 10.9 [2.42–48.96] and 4.38 [1.45–13.24] for CV and all-cause mortalities, respectively). The hemodialysis patients with CD16+ monocyte level in a low but mostly covering normal range also portended a poor prognosis. The findings shed some light for nephrologists on future prospects of early recognizing immune dysfunction and improving early intervention outcomes.

## 1. Introduction

It has been established beyond any doubt that cardiovascular (CV) events are an important cause of death, accounting for up to 40–50%, in end-stage renal disease (ESRD) patient population. In the early 70s, Foley et al. have reported that mortality from cardiovascular disease (CVD) is 10–20 times higher in ESRD patients compared with the general population [1]. Interestingly, some authors found that mortality from non-CV disease in dialysis patients was also increased to the same extent as mortality from CVD [2, 3]. Over these years, the potential link between CV and non-CV mortality was explored. Ishani et al. [4] showed that septicemia or bacteremia in dialysis patients was associated with subsequent CV-related events such as myocardial infarction, heart failure, and stroke. On the other hand, the risk of myocardial infarction and that of stroke were substantially higher after a diagnosis of systemic respiratory tract infection [5]. These studies suggested that both CV and infectious causes of death are linked to inflammation, and possibly, these two events may aggravate each other.

Mounting evidence shows that disturbed endothelial function may be an early marker of atherosclerotic process [6]. Clinical and experimental data support a link between endothelial dysfunction and inflammation [7–10]. Chronic systemic inflammation, a common feature in dialysis patients, has been identified as an epidemiologically important risk factor for CV morbidity and mortality in dialysis patients [11–13]. Of 30 prevalent patients, 50% had elevated serum levels of inflammatory markers such as C-reactive protein, IL-6, and procalcitonin [12, 14, 15]. In addition, a shift towards proinflammatory monocyte subsets [16] and monocyte dysfunction [17] is also noted in these patients. Available evidence showed that even low-grade systemic inflammation has been found to be associated with devastating prognosis of dialysis patients [18–21].

Monocytes can be subdivided into three phenotypically and functionally distinct subpopulations based on the expression of the lipopolysaccharide (LPS) receptor (CD14) and the CD16 (Fcgamma receptor III) [22, 23]. In healthy individuals, approximately 80–90% of monocytes are highly CD14 positive and CD16 negative (CD14++CD16−): classical monocytes. The remaining 10–20% of monocytes are CD16 positive, which are further subdivided into CD14++CD16+ and CD14+CD16++ cells, intermediate and nonclassical monocytes, respectively [23]. Compared with CD16 negative conventional monocytes, CD16 positive monocytes, also called proinflammatory monocytes, express higher levels of major histocompatibility complex (MHC) class II antigens, adhesion molecules, chemokine receptors, and proinflammatory cytokines such as TNF-*α*, but lower levels of the anti-inflammatory cytokine, that is, IL-10 [24, 25]. CD16 positive monocytes are elevated in various pathologic conditions, including inflammatory and infectious diseases [26], cancer [27], and in coronary heart disease as ESRD [16, 28, 29]. However, to date, the mechanism by which CD16 positive monocytes increase remains unclear.

Here, we examined the interrelationships between the proportion of proinflammatory monocytes (CD16+ monocytes) and all-cause mortality as well as CV mortality in a cohort of stable ESRD patients on hemodialysis. This study might shed more light on the potential mechanisms that link microinflammation with future CV events.

## 2. Methods

### 2.1. Patients and Study Sample

Adult outpatients on hemodialysis at the Tungs' Taichung MetroHarbor Hospital (TTMHH) in June 2009 were enrolled. A total of 136 patients were eligible. All the enrolled patients signed informed consents. This study was conducted in full compliance with the provisions of the Personal Information Protection Act and the Human Subjects Research Act of Taiwan and was approved by the institutional review board (number: 102011).

All the patients were dialyzed three times a week with a high-flux polysulfone membrane (FX80 and FX100; Fresenius Medical Care, Bad Homburg, Germany) and bicarbonate dialysate solutions. The median blood flow rate was 280 ml/min (range 250–300 ml/min). All dialysate flows were 800 ml/min, and treatment time was 240 minutes for each patient. All patients were dialyzed through a native arteriovenous (AV) fistula. Blood samples were obtained just before the midweek dialysis session. The dialysate revealed concentrations of bacterial and endotoxin contamination below the detection limit (100 colony-forming units/ml and <0.25 endotoxin units). Systolic and diastolic blood pressures (SBP and DBP) were measured in a supine position and after at least a 10-minute rest using a full automatic noninvasive sphygmomanometer.

Each patient's medical chart prior to study enrollment was thoroughly reviewed, and data pertaining to underlying kidney disease, history of CVD, and common comorbid conditions were extracted. The causes of renal failure were diabetic nephropathy (*n* = 68), chronic glomerulonephritis (*n* = 30), polycystic kidney disease (*n* = 3), hypertensive nephrosclerosis (*n* = 15), or unknown (*n* = 20). Patients who had started on hemodialysis for less than 3 months had history of chronic liver diseases, neoplasm, or inflammatory diseases, and those on long-term corticosteroids were excluded. A preexisting history of CVD was defined as a history of coronary artery disease (CAD, including a history of myocardial infarction, coronary artery angioplasty/stenting/bypass surgery, and carotid endarterectomy/stenting), cerebrovascular disease (CeVD, e.g., stroke), nontraumatic lower extremity amputation, and lower limb artery bypass surgery/angioplasty/stenting. Diabetes mellitus (DM) cases were ascertained if a patient had a history of DM diagnosis, a spontaneous plasma glucose level of >200 mg/dl, and/or received hypoglycemic treatment. The survival data were then retrieved in September 2016.

### 2.2. Laboratory Methods

All blood samples were collected during the midweek dialysis from the AV fistula, immediately after the insertion of the dialysis cannula but before the administration of heparin. Blood was sampled in 4 c.c. Venoject II tubes and centrifuged (10 min, 3000 rpm) and stored at −70°C pending analyses, if not analyzed immediately. Serum albumin, urea, creatinine (Cr), total cholesterol, and triglyceride (TG) were determined according to standard methods. The serum levels of high-sensitivity C-reactive protein (hsCRP) were measured using a Behring Nephelometer II (Dade Behring, Tokyo, Japan).

### 2.3. Determination of CD14 and CD16 Mononuclear Phenotype

Peripheral blood was collected by venipuncture using ethylenediaminetetraacetic acid (EDTA) as an anticoagulant. For cytometric analysis, monoclonal antibodies against CD14 (fluorescein isothiocyanate (FITC) conjugated; clone RMO52; Beckman Coulter, Miami, FL, USA), CD16 (phycoerythrin (PE) conjugated; clone 3G8; Beckman Coulter, Miami, FL, USA), CD45 (phycoerythrin cyanin-5 (PC5); clone J33; Beckman Coulter, Miami, FL, USA), and CD56 (clone IM2073; Beckman Coulter, Miami, FL, USA) were used. Briefly, 100 l of the whole blood was stained with saturating amounts of the abovementioned monoclonal antibodies and corresponding isotype controls. After incubation for 15 min at room temperature in the dark according to the manufacturer's recommendations, OptiLyse C (Beckman Coulter, Miami, FL) was added to lyse RBC and the samples were fixed. Fixed cells were analyzed by flow cytometry within 6 hours.

Determination of leukocyte and monocyte subset distribution was performed using a FC500-Cytometer (Beckman Coulter), and CXP analysis software (version 2.2) was used (Schroers et al., 2005). Monocytes were identified as CD45 positive and CD56 negative cells exhibiting a specific forward and sideward scatter profile. Monocytes were then gated in an SSC/CD dot plot, identifying monocytes as CD86 cells with monocyte scatter properties. Subsets of CD14 monocytes with and without CD16 were defined according to the surface expression pattern of the lipopolysaccharide receptor CD14 and the CD16 (Fcgamma receptor III). One million cells were analyzed from each sample, and the percentage of CD16 positive mononuclear cells (CD14+/CD16+ and CD14++/CD16+) and the number of cells out of the total monocytes were compared using fluorescent microbeads (Flow-Count, Beckman Coulter). The CD86 antibody (clone HA5.2B7; Beckman Coulter, Miami, FL, USA) was used in this study.

### 2.4. Statistical Analysis

The sample characteristics were summarized using frequencies and percentage for categorical variables and using median (i.e., the second quartile, *q*_2_), interquartile interval (IQI, an interval bounded by the first and the third quartiles, *q*_1_ and *q*_3_), mean, and standard deviation (SD) for continuous variables. Spearman's correlation analysis was applied to evaluate bivariate associations between CD16+ monocytes and other observed variables. The Cox regression was applied to evaluate the association of mortality with CD16+ monocytes and with other variables. Two types of mortalities were investigated in this study: CV and all-cause mortalities. The starting point of the survival time was designed at 2009/06/01. Cases who survived till 2016/09/01, transferred to other centers or transplanted during study observation period, were censored at the date. The raw CD16+ monocytes were categorized according to its three quartiles (*q*_1_, *q*_2_, and *q*_3_) into a variable of four levels (from the lowest to the highest level: Q1, Q2, Q3, and Q4). The Cox regression analysis results were displayed in hazard ratio (HR), its associated 95% confidence interval (CI), and *p* value. The crossover pattern of hazards among the four-level CD16+ monocytes was modeled using time-dependent effect in the Cox regression model. Throughout this study, tests for statistical associations were evaluated at a significance level of 0.05. The analyses were all performed in SAS version 9.1.

## 3. Results

The descriptive statistics of the whole study sample were summarized in Table 1. Of the 136 patients, 39 died in CVD, 18 died in other causes, 8 censored because of transplantation or transferred to another center, and 71 survived till the follow-up ends. The mean (minimum–maximum) follow-up time was 5.57 (0.10–7.25) years for the overall sample and was 7.03 (2.66–7.25) years for the 79 nondeath cases.

**Figure 1:**
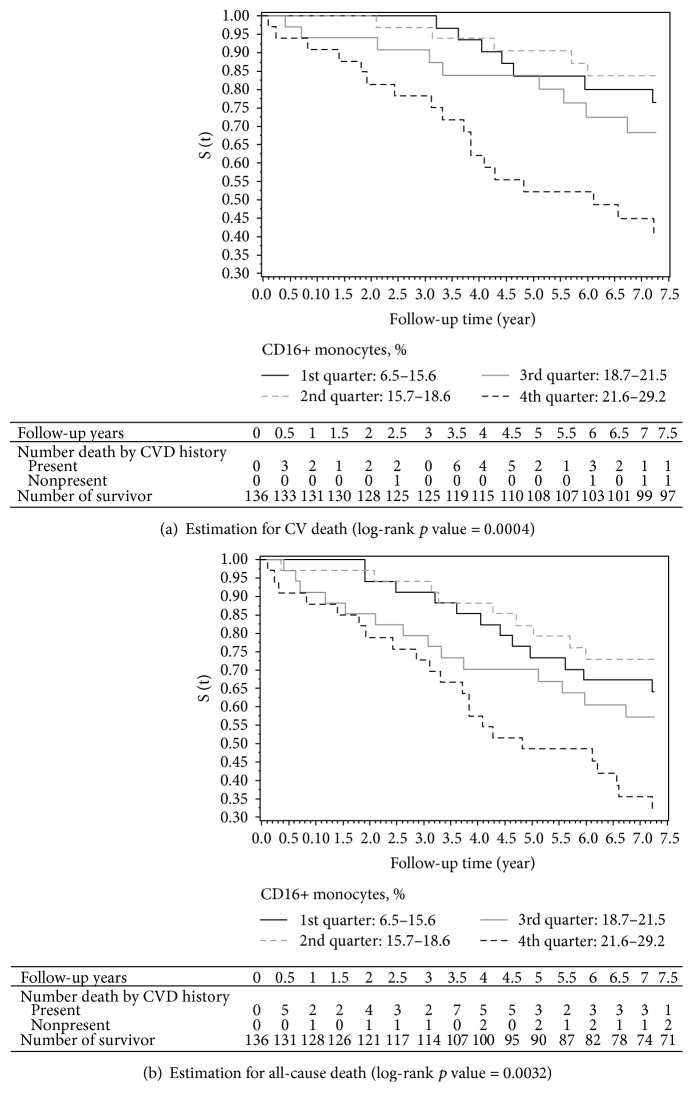
The Kaplan-Meier curves by CD16+ monocyte level. The Kaplan-Meier curves for CD16+ monocyte level within the lowest to the highest quarters were indicated by black solid line, gray dashed line, gray solid line, and black dashed line. The CD16+ monocyte ranges of the four quarters were the same as those listed in the second row of Table 1. The numbers of death by baseline CVD status and survivor during follow-up were listed below the figures.

The Kaplan-Meier curves of CV death and of all-cause death by CD16+ monocyte level were displayed in Figure 1, where follow-up details on the observed case numbers were listed below the figures. The curves overall appeared that patients with CD16+ monocytes in the fourth quarter had the worst survival rate (the black dotted line in Figure 1) and those in the second quarter had better survival rate (the gray dashed line in Figure 1) compared to others. Patients without preexisting CVD history accounted a minor proportion in the overall death numbers: 10.26% (4/39) for CV death and 23.08% (15/65) for all-cause death. Such numbers were reduced to 5.41% (2/37) and 12.73% (7/55) in subsequent Cox regression analyses because of missing covariates. Since the survival curves for CD16+ monocytes in the first and second quarters were a crossover at 3.5 years, the time-dependent effect between the two levels of CD16+ monocytes was incorporated in the later Cox regression for CVD and all-cause mortalities.

The bivariate analysis for CD16+ monocytes and other variables was displayed in Table 2. Most variables were not significantly associated with CD16+ monocyte level, except for age, ferritin, and preexisting CVD history at baseline. Patients having preexisting CVD tended to have a high-level CD16+ monocytes (*p* < 0.0001). The Spearman correlation coefficients (*p* value) for CD16+ monocytes with ferritin and with age was, respectively, 0.18 (0.0412) and 0.25 (0.0039). Both manifested that higher CD16+ monocytes were correlated to higher ferritin and older age.

The univariate Cox regression analysis results in Table 3 showed that patients with medical conditions such as DM, hypertension, and preexisting CAD or CeVD history tended to have higher risk in both CV death and all-cause death. In specific, patients with DM at baseline had significantly higher CVD death risk (HR = 2.18, 95% CI: 1.12–4.25) and those with a preexisting CeVD event had significantly higher all-cause death risk (HR = 2.05, 95% CI: 1.14–3.67). Patients of old age and with high level of hsCRP, fasting blood sugar (FBS), and glycated hemoglobin (HbA1c) tended to have higher risk in both mortalities (all these variables had HRs > 1 in Table 3). Those with high level in albumin and Cr tended to have lower risk in both mortalities (all these variables had HRs < 1 in Table 3). For instance, a CVD death risk HR value of 1.03 (95% CI: 1.01–1.06) for the variable age in Table 3 indicated that a 1-year increment in age is significantly associated with 3% increase in CVD death risk. An all-cause death risk HR value of 0.13 (95% CI: 0.04–0.41) for the variable albumin indicated that 1 g/dl increment in albumin is significantly associated with 87% decrease in all-cause death risk. Patients who had CD16+ monocyte level lying in the second quarter (i.e., CD16+ monocyte level > *q*_1_ and CD16+ monocyte level ≤ *q*_2_) expressed the lowest risks in both CVD and all-cause death (a J-shaped relationship).

Both the Kaplan-Meier curve and the univariate Cox regression analysis demonstrated a J-shaped relationship between CD16+ monocytes and patients' death risks, especially after the time of follow-up exceeds 3.5 years. Considering the possible effect of the lowest quartile of CD16+ monocytes and the risk of death, any possible reverse causation was adequately addressed in the analyses by maintaining a varying reference category. A multiple Cox regression analysis (Table 4) demonstrated that the J-shaped relationship between CD16+ monocytes and hemodialysis patients' death risks persisted after accounting for baseline conditions, for the hazard crossover effect between the two lowest quarters of CD16+ monocytes, and for a range of covariates. Patients with CD16+ monocytes in the fourth quarter manifested significantly higher death risks as compared to all other quarters; the HRs ranged from 2.83 to 30.85 for CV death and from 1.21 to 5.84 for all-cause death after adjusting other covariates (see Table 4 for detailed results). Further, patients with CD16+ monocytes below *q*_1_ had an elevated adjusted HR for both CV death (HR = 10.9, *p* = 0.002) and for all-cause death (HR = 4.38, *p* = 0.009) in the fully adjusted model.

In analysis regarding CV death risk, preexisting CeVD and CAD history had a significant effect after adjusting the effect of CD16+ monocytes (HR = 6.98 and HR = 44.57, resp., both *p* ≤ 0.001). Interestingly, a preexisting CAD history appeared to be associated with higher CV death risk than a preexisting CeVD (HR = 6.39, *p* = 0.004). Patients of old age (above median), with PLT, SBP, and cholesterol above median, with Cr below the first quartile, with uric acid (UA) and DBP out of the IQI, and with ratio of TG to total cholesterol (rTG) below the third quartile, were associated with higher CV death risk. For all-cause death risk, the presence of preexisting CeVD and CAD had a significant effect (HR = 2.74 and HR = 9.4, resp., *p* = 0.003 and *p* < 0.001) after adjusting the effect of CD16+ monocytes. Patients of age above median, with PLT above median, with Cr below median, with UA and DBP out of the IQI, and with rTG and ferritin below the third quartile, were associated with higher all-cause death risk. The detailed results were listed in Table 4.

## 4. Discussion

Our data accord with some previous findings of increased mortality in dialysis patients with higher percentages of nonclassical CD16 positive monocytes. Recent studies have established that phenotypic variations in the surface of monocytes are associated with the occurrence of CVD in both chronic kidney disease (CKD) and non-CKD patients. While Berg et al. found that classical CD16 negative monocytes can predict future CV risk in nonuremic population [30], some authors found that intermediate CD14++CD16+ monocytes predict CV events in CKD patients [31, 32]. Differences in study design and studied populations may account for some of the discrepancies regarding the correlation of monocyte subsets and adverse cardiac events in these studies. Nevertheless, flow cytometry is a powerful technique and its use has obviously allowed for risk stratification in a wide variety of diseases.

The innate immune system plays a major role in the initiation and propagation of atherosclerosis, with monocytes/macrophages being the key component in this process [33]. Apart from being responsible for counteracting exogenous bacterial, viral, and fungal infections [34], they are also involved in endogenous inflammatory processes. They contribute to atherogenesis through promoting leukocyte recruitment to plaques, and their roles are also mediated by activation of downstream signaling pathways, such as nuclear factor kappa-B pathway [35]. Monocyte involvement in the development of atherosclerotic plaques was reported in the 1970s, with monocyte accumulation demonstrated in porcine atherosclerotic lesions [36]. In recent years, we became aware of the role of different monocyte subsets in the pathogenesis of atherosclerosis, particularly specific monocyte subpopulations with their diverse phenotypes and sentinel roles in both the innate and adaptive immune system. Our understanding of how monocyte subsets participate in this process is largely based on mouse models of atherosclerosis [37, 38].

In non-CKD populations, many cohort and case-control studies have documented an association of monocytosis with cardiovascular diseases [39–42]. Elevated monocyte counts were also identified as an independent predictor of total and CV mortality in hemodialysis patients [43]. In a cohort of 951 patients, Rogacev et al. [31] found that nonclassical CD14+CD16+ monocytes independently predicted cardiovascular events in subjects referred for elective coronary angiography. Numbers of CD16 positive monocytes but not overall monocyte counts positively correlate with body mass index and insulin resistance as well as diabetes and intima-media thickness [44]. In patients with symptomatic CAD compared to healthy controls, the percentage of CD16 positive monocytes was found to be increased after adjustment for common risk factors [45, 46]. Assessment of plaque vulnerability in patients with both stable and unstable angina pectoris found that more vulnerable plaques were associated with an increase in percentage of CD16 positive monocytes.

CKD had been shown to alter the number, subset distribution, and function of circulating monocytes [47, 48]. In previous studies [16, 31, 32], patients with CKD have an increased percentage of CD16 positive monocytes in the circulation. In our study, we further observed that in patients with preexisting CVD, the presence of higher percentage of CD16 positive monocytes was found to be associated with increased CV and all-cause death.

More interestingly, we found that a subset of dialysis patients with CD16+ monocytes falling within the normal range tends to suffer great risk of CV death. Advanced CKD is characterized by the dynamic coexistence of the generalized immune depression that contributes to the high prevalence of infections among these patients and systemic inflammation that may contribute to CVD. Accumulation of proinflammatory cytokines may be due to decreased renal elimination and/or increased generation following induction by various factors such as uremic toxins, oxidative stress, volume overload, and comorbidities [49, 50]. ESRD is associated with immunosuppression due to the impact of the uremic milieu and a variety of associated metabolic disorders on the other. Impaired monocyte function, including defects in chemotaxis, phagocytosis, and a decrease in the production of cytokines, had been reported [50]. Frequent exposure to these diverse external stimuli might lead to a state of chronic low-grade activation, and a high-percentage monocytic primed cell was found in hemodialysis patients [51].

The prevailing and continuous antigenic stimulation might result in exhaustion in the downstream signaling cascade, and this might subsequently impair the innate and adaptive components of the immune system's response to microbial challenge. The presence of a subgroup of our patients with functional monocyte deactivation may be due to LPS tolerance. This state of “immune paralysis” in these patients may be related to downregulation of toll-like receptor, especially toll-like receptor-2 (TLR-2) and toll-like receptor-4 (TLR-4) expression on monocytes [49, 52]. TLR-2 and TLR-4 are involved in innate immunity, and activation of these receptors leads to systemic inflammation in the host. Several authors found that a decrease of TLR-4 was found on unstimulated monocytes in CKD patients compared with healthy controls [52–54]. The etiological factor of “immune paralysis” may be related to chronic endotoxemia [55], frequent blood membrane interaction, or other toxic metabolites related to uremic milieu. It seems possible that continuous activation of monocytes suppresses the expression of TLR-4, contributing to immune deficiency and increased incidence and severity of infections in ESRD population. Clearly, the J-shaped effect of low CD16+ monocytes on CV death risk observed in this study needs further research for it to be clarified.

Lastly, the analysis results of this study, namely, the association between CD16+ monocytes and mortalities in hemodialysis patients, were obtained mostly in patients with a preexisting CVD history. In the multiple Cox regression analysis, we found that those without CVD history at baseline had just 2 nonmissing cases suffered from CV death and 7 nonmissing cases from all-cause death. Moreover, the association pattern shown in Table 4 remained unchanged when the analyses were performed on the sample composed of those with CVD history at baseline.

We acknowledge several limitations of this study. One of them is the relatively small number of patients, particularly the relatively small number of patients without preexisting CAD. Thus, caution should be exercised in the interpretation of our results. Besides, our patient population was also limited to those on hemodialysis and may not be generalizable to the broader population.

Taken together, the results of this study indicated that high level in CD16+ monocytes was associated with significantly higher risks in CV and all-cause death in hemodialysis patients with preexisting CAD. Overall, nonclassic monocytes were detrimental, whereas the minor subset of the relatively low CD16-expressing monocytes was associated with an unfavorable clinical outcome. In spite of the limited study sample, we highlight the current facts and future perspectives of how the assessment of microinflammation can assist clinicians in early and efficient recognition of inappropriate performance of the immune system to reduce mortality. Nevertheless, more studies based on large-scale cohort are still desired to elucidate this issue further.

## Figures and Tables

**Table 1:** The summary of the sample characteristics (*n* = 136).

Variables	Missing number	*n* (%)
Sex, female versus male	0	62 (45.59) versus 74 (54.41)
DM, no versus yes	0	68 (50) versus 68 (50)
Hypertension, no versus yes	0	38 (27.94) versus 98 (72.06)
Preexisting CVD, no versus yes	3	62 (46.62) versus 71 (53.38)
HD vintage, year	0	
0–3		20 (14.71)
>3, ≤5		37 (27.21)
>5, ≤8		37 (27.21)
>8		42 (30.88)
		*Median (IQI)*
CD16+ monocytes, %	1	18.6 (15.6, 21.5)
HD vintage, year	0	6.04 (3.92, 9.04)
Age, year	0	59 (51.5, 69)
BMI, kg/m^2^	0	23.62 (21.08, 25.32)
WBC, 10^3^/mm^3^	1	6.6 (5.4, 7.5)
Monocyte, 10^3^/*μ*l	1	5.9 (4.8, 7)
AbsoMono, cells/*μ*l	1	367.2 (291.2, 482.4)
HsCRP, mg/l	2	2.8 (1.5, 3.8)
Hb, g/dl	1	11.2 (10.1, 12.2)
PLT, 10^4^/cm^3^	1	160 (54, 218)
FBS, mg/dl	1	96 (82, 139)
HbA1c	2	6 (5, 7.1)
Albumin, g/dl	1	4.2 (4, 4.4)
Ferritin, *μ*g/dl	2	679 (467, 838)
TG, mg/dl	1	115 (81, 187)
HDL, mg/dl	2	43.5 (34, 56)
Cholesterol, mg/dl	3	163 (140, 189)
rTG	3	0.77 (0.48, 1.13)
rHDL	3	0.28 (0.21, 0.36)
cHDL	3	116 (94, 142)
BUN, mg/dl	2	66.5 (58.1, 75.7)
Cr, mg/dl	2	10.5 (9.2, 11.9)
UA, mg/dl	2	7.6 (6.7, 8.5)
Ca, mg/dl	2	9.4 (9, 9.8)
P, mg/dl	3	4.6 (3.8, 5.6)
SBP, mmHg	0	138.5 (121.5, 154.5)
DBP, mmHg	0	77 (69, 84.5)

Note: the numbers in the second column indicated the missing numbers of each variable. DM: diabetes mellitus; CVD: cardiovascular diseases including CAD and CeVD; CAD: coronary artery diseases; CeVD: cerebrovascular disease; BMI: body mass index; WBC: white blood cell count; AbsoMono: absolute monocyte; HsCRP: high-sensitivity C-reactive protein; Hb: hemoglobin/haemoglobin; PLT: platelet count; FBS: fasting blood sugar; HbA1c: glycated hemoglobin; TG: triglyceride; HDL: high-density lipoprotein cholesterol; cholesterol: total cholesterol; rTG: ratio of TG to cholesterol; rHDL: ratio of HDL to total cholesterol; cHDL: the value resulted from subtracting the level of HDL from the level of total cholesterol; BUN: blood urea nitrogen; Cr: creatinine; UA: uric acid; Ca: serum calcium; P: serum phosphorus; SBP: systolic blood pressure; DBP: diastolic blood pressure; IQI: interquartile interval which is bounded by the first and the third quartiles (*q*_1_ and *q*_3_) of the variable listed in the first column.

**Table 2:** The results of association analysis for CD16+ monocytes.

Variables	CD16+ monocyte level				*p* value
	6.5~15.6	>15.6, ≤18.6	>18.6, ≤21.5	>21.5, ≤29.2	
*n*	34	34	34	33	
	*n* (%)	*n* (%)	*n* (%)	*n* (%)	
Sex					
Female	15 (44.12%)	14 (41.18%)	15 (44.12%)	18(54.55%)	0.374
Male	19 (55.88%)	20 (58.82%)	19 (55.88%)	15(45.45%)	
DM					
No	20 (58.82%)	18 (52.94%)	16 (47.06%)	13(39.39%)	0.083
Yes	14 (41.18%)	16 (47.06%)	18 (52.94%)	20(60.61%)	
Hypertension					0.806
No	8 (23.53%)	11 (32.35%)	11 (32.35%)	8(24.24%)	
Yes	26 (76.47%)	23 (67.65%)	23 (67.65%)	25(75.76%)	
Preexisting CAD or CeVD					
No	22 (64.71%)	20 (58.82%)	13 (40.63%)	7(21.21%)	<0.0001^∗^
Yes	12 (35.29%)	14 (41.18%)	19 (59.38%)	26(78.79%)	
HD vintage (year)					
0–3	7 (20.59%)	4 (11.76%)	6 (17.65%)	3(9.09%)	0.849
>3, ≤5	8 (23.53%)	10 (29.41%)	8 (23.53%)	11(33.33%)	
>5, ≤8	6 (17.65%)	11 (32.35%)	14 (41.18%)	6(18.18%)	
>8	13 (38.24%)	9 (26.47%)	6 (17.65%)	13(39.39%)	
	*Mean ± SD*	*Mean ± SD*	*Mean ± SD*	*Mean ± SD*	
CD16+ monocytes, %	12.77 ± 2.32	16.96 ± 0.87	20.28 ± 0.87	24.77 ± 2.07	—
HD vintage, year	7.99 ± 5.74	6.8 ± 3.81	6.29 ± 3.82	6.89 ± 3.59	0.753
Age, year	56.03 ± 12.03	59.91 ± 11.35	61.59 ± 13.15	63.45 ± 10.67	0.004^∗^
BMI, kg/m^2^	23.1 ± 3.38	23.31 ± 3.64	23.1 ± 3.27	25.03 ± 3.88	0.238
WBC, 10^3^/mm^3^	7.08 ± 2.12	6.28 ± 1.43	6.53 ± 1.58	6.32 ± 1.35	0.245
Monocyte, 10^3^/*μ*l	5.71 ± 1.77	5.89 ± 1.58	6.27 ± 1.74	6.06 ± 1.47	0.192
AbsoMono, cells/*μ*l	413.94 ± 214.01	366.77 ± 117.41	408.65 ± 149.62	380.39 ± 120.19	0.961
HsCRP, mg/l	2.57 ± 1.42	2.42 ± 1.46	2.49 ± 1.41	2.96 ± 1.34	0.367
Hb, g/dl	11.47 ± 1.59	11.39 ± 1.31	11.15 ± 1.57	10.97 ± 1.27	0.135
PLT, 10^4^/cm^3^	143.21 ± 90.39	124.96 ± 82.61	163.69 ± 88.49	153.32 ± 89.62	0.311
FBS, mg/dl	110.85 ± 42.25	111.86 ± 43.94	126.56 ± 59.31	114.76 ± 45.17	0.732
HbA1c, %	6.38 ± 1.72	5.96 ± 1.29	6.29 ± 1.33	6.59 ± 1.71	0.198
Albumin, g/dl	4.24 ± 0.22	4.43 ± 1.45	4.18 ± 0.32	4.1 ± 0.29	0.163
Ferritin, *μ*g/dl	657.41 ± 256.8	594.68 ± 271.22	745.94 ± 326.2	717.58 ± 288.81	0.041^∗^
TG, mg/dl	144.56 ± 107.83	132.05 ± 73.32	154.29 ± 105.66	141.42 ± 83.4	0.782
HDL, mg/dl	47.18 ± 17.99	48.82 ± 17.11	48.03 ± 19.79	43.48 ± 14.52	0.877
Cholesterol, mg/dl	168.5 ± 37.08	165.7 ± 37.16	161.24 ± 37.81	164.12 ± 41.12	0.767
rTG	0.86 ± 0.6	0.82 ± 0.39	0.9 ± 0.52	0.88 ± 0.49	0.805
rHDL	0.29 ± 0.11	0.31 ± 0.12	0.3 ± 0.11	0.27 ± 0.09	0.757
cHDL, mg/dl	121.32 ± 36.73	116.58 ± 37.82	113.21 ± 34.76	120.64 ± 38.17	0.689
BUN, mg/dl	67.6 ± 14.69	67.32 ± 12.52	66.88 ± 12.84	65.98 ± 15.42	0.59
Cr, mg/dl	10.84 ± 2	10.66 ± 2.24	10.72 ± 2.3	10.07 ± 2.12	0.149
UA, mg/dl	7.56 ± 1.49	7.85 ± 1.35	7.65 ± 1.25	7.17 ± 1.93	0.622
Ca, mg/dl	13.32 ± 22.21	9.43 ± 0.39	9.26 ± 0.65	9.38 ± 0.79	0.225
P, mg/dl	4.65 ± 1.46	4.75 ± 1.35	4.41 ± 1.2	4.76 ± 1.4	0.55
SBP, mmHg	134.91 ± 22.34	138.85 ± 19.65	135.79 ± 15.02	140.88 ± 25.02	0.446
DBP, mmHg	78 ± 10.53	76.38 ± 10.38	76.15 ± 8.33	75.79 ± 9.69	0.479

Note: the abbreviations are the same as those denoted in Table 1. ^∗^*p* value of Spearman's association test using the raw data value of CD16+ monocytes and observed variables.

**Table 3:** The univariate Cox regression analysis results for death risk.

Outcome types	CVD				All causes			
Covariates	HR	95% CI		*p* value	HR	95% CI		*p* value
CD16+ monocytes, %								
Q1_(≤3.5 y)_ versus Q2	0.45	0.05	3.96	0.469	0.92	0.27	3.09	0.888
Q1_(>3.5 y)_ versus Q2	5.16	0.56	47.86	0.149	2.00	0.53	7.63	0.308
Q3 versus Q2	2.18	0.73	6.52	0.161	1.86	0.81	4.31	0.145
Q4 versus Q2	5.13	1.90	13.84	0.001^∗^	3.44	1.58	7.48	0.002^∗^
Sex, male versus female	0.96	0.51	1.79	0.888	0.91	0.54	1.53	0.72
Age, years	1.03	1.01	1.06	0.011^∗^	1.04	1.02	1.06	<0.001^∗^
HD vintage, years	0.97	0.9	1.04	0.42	0.95	0.89	1.01	0.132
DM, yes versus no	2.18	1.12	4.25	0.021^∗^	1.87	1.09	3.2	0.023^∗^
Hypertension, yes versus no	3.14	1.23	8.04	0.017^∗^	2.16	1.09	4.27	0.027^∗^
Preexisting CAD, yes versus no	7.99	3.63	17.59	<0.001^∗^	5.66	3.11	10.3	<0.001^∗^
Preexisting CeVD, yes versus no	2.11	1.04	4.29	0.038^∗^	2.05	1.14	3.67	0.016^∗^
BMI, kg/m^2^	1.03	0.94	1.12	0.535	0.98	0.91	1.05	0.547
WBC, 10^3^/*μ*l	1.16	0.97	1.4	0.112	1.13	0.97	1.32	0.113
Monocyte,10^3^/*μ*l	1.06	0.87	1.28	0.558	1.05	0.89	1.23	0.583
HsCRP, mg/l	1.49	1.15	1.91	0.002^∗^	1.38	1.12	1.68	0.002^∗^
FBS, mg/dl	1.01	1	1.01	0.019^∗^	1.01	1	1.01	0.01^∗^
rHDL	4.98	0.31	80.71	0.259	4.95	0.5	48.94	0.172
cHDL, mg/dl	4.98	0.31	80.71	0.259	4.95	0.5	48.94	0.172
HbA1c, %	1.26	1.05	1.52	0.015^∗^	1.21	1.04	1.42	0.015^∗^
P, mg/dl	1.06	0.84	1.34	0.606	1.01	0.83	1.22	0.945
SBP, mmHg	1.01	1	1.03	0.171	1.01	1	1.02	0.181
DBP, mmHg	1.01	0.98	1.05	0.43	1.01	0.98	1.04	0.521
AbsoMono, cells/*μ*l	1	1	1	0.121	1	1	1	0.127
PLT, 10^3^/*μ*l	1	1	1.01	0.042^∗^	1	1	1.01	0.133
Ferritin, *μ*g/dl	1	1	1	0.155	1	1	1	0.761
TG, mg/dl	1	1	1	0.675	1	1	1	0.334
HDL, mg/dl	1	0.98	1.02	0.978	1	0.98	1.01	0.84
Cholesterol, mg/dl	0.99	0.98	1	0.121	0.99	0.98	1	0.029^∗^
rTG	0.86	0.44	1.68	0.654	0.82	0.46	1.43	0.48
Hb, g/dl	0.89	0.7	1.13	0.348	0.92	0.76	1.12	0.405
Albumin, g/dl	0.23	0.06	0.85	0.028^∗^	0.13	0.04	0.41	<0.001^∗^
BUN, mg/dl	1	0.97	1.02	0.802	0.99	0.97	1.01	0.165
Cr, mg/dl	0.83	0.71	0.97	0.019^∗^	0.77	0.67	0.88	<0.001^∗^
UA, mg/dl	0.89	0.72	1.1	0.274	0.86	0.73	1.01	0.073
Ca, mg/dl	0.97	0.8	1.17	0.734	0.96	0.76	1.21	0.732

Note: the abbreviations are the same as those denoted in Table 1. Q1, Q2, Q3, and Q4 denoted the four ascending classes of the categorical variable which was derived from categorizing each covariate by its three quartiles. The values of the three quartiles were listed in Table 1. Rows indicated as Q1_(≤3.5 y)_ versus Q2 and Q1_(>3.5 y)_ versus Q2 listed the time-varying effect of CD16+ monocytes below the lowest quartile for follow-up time before and after 3.5 years. ∗ indicates *p* values of less than 0.05.

**Table 4:** The multiple Cox regression analysis results for death risk.

Outcome types	CVD death				All-cause death			
Covariates	HR	95% CI		*p* value	HR	95% CI		*p* value
CD16+ monocytes, %								
Q4 versus Q3	12.81	3.72	44.09	<0.001^∗^	3.26	1.49	7.14	0.003^∗^
Q4 versus Q2	30.85	7.12	133.8	<0.001^∗^	5.28	2.07	13.49	<0.001^∗^
Q3 versus Q2	2.41	0.58	10.03	0.227	1.62	0.61	4.29	0.333
Follow-up time ≤ 3.5 years								
Q1 versus Q2	1.63	0.15	17.33	0.685	0.9	0.26	3.16	0.875
Q4 versus Q1	18.92	2.17	164.9	0.008^∗^	5.84	1.71	19.98	0.005^∗^
Q3 versus Q1	1.48	0.16	13.47	0.73	1.79	0.52	6.17	0.358
Follow-up time > 3.5 years								
Q1 versus Q2	10.9	2.42	48.96	0.002^∗^	4.38	1.45	13.24	0.009^∗^
Q4 versus Q1	2.83	0.81	9.86	0.102	1.21	0.44	3.29	0.716
Q3 versus Q1	0.22	0.05	0.91	0.037^∗^	0.37	0.13	1.07	0.067
Baseline medical condition								
CeVD history, yes versus no	6.98	2.18	22.3	0.001^∗^	2.74	1.41	5.32	0.003^∗^
CAD history, yes versus no	44.57	13.1	151.7	<0.001^∗^	9.4	4.53	19.48	<0.001^∗^
CAD history versus CeVD history	6.39	1.79	22.8	0.004^∗^	3.43	1.34	8.75	0.01^∗^
Age, >*q*_2_ versus others	2.88	1.14	7.28	0.025^∗^	2.36	1.24	4.52	0.009^∗^
Cholesterol, >*q*_2_ versus others	2.98	1.19	7.44	0.02^∗^	—	—	—	—
Platelet, >*q*_2_ versus others	7.23	2.66	19.67	<0.001^∗^	3.99	2.04	7.79	<0.001^∗^
Cr								
≤*q*_1_ versus others	4.26	1.82	10	<0.001^∗^	—	—	—	—
≤*q*_2_ versus others	—	—	—	—	4.49	2.31	8.75	<0.001^∗^
UA, ≤*q*_1_ or >*q*_3_ versus others	7.38	2.47	22.01	<0.001^∗^	2.5	1.35	4.62	0.003^∗^
SBP, >*q*_2_ versus others	5.11	1.92	13.59	0.001^∗^	—	—	—	—
DBP, ≤*q*_1_ or >*q*_3_ versus others	7.43	2.62	21.06	<0.001^∗^	4.08	1.98	8.4	<0.001^∗^
rTG, >*q*_3_ versus others	0.18	0.07	0.47	<0.001^∗^	0.21	0.09	0.47	<0.001^∗^
Ferritin, >*q*_3_ versus others	—	—	—	—	0.45	0.23	0.87	0.018^∗^

Note: the abbreviations are the same as those indicated in Table 1. *q*_1_, *q*_2_, and *q*_3_ denoted the three quartiles—the first quartile, median, and the third quartile—of each covariate and the values were listed in Table 1. Q1, Q2, Q3, and Q4 here denoted the four ascending categories derived from the categorized CD16+ monocyte levels by its three quartiles. ∗ indicates *p* values of less than 0.05.
